# Exploring the Perioperative Use of DOACs, off the Beaten Track

**DOI:** 10.3390/jcm13113076

**Published:** 2024-05-24

**Authors:** Fabiana Lucà, Fabrizio Oliva, Simona Giubilato, Maurizio Giuseppe Abrignani, Carmelo Massimiliano Rao, Stefano Cornara, Giorgio Caretta, Stefania Angela Di Fusco, Roberto Ceravolo, Iris Parrini, Adriano Murrone, Giovanna Geraci, Carmine Riccio, Sandro Gelsomino, Furio Colivicchi, Massimo Grimaldi, Michele Massimo Gulizia

**Affiliations:** 1 Cardiology Department, Grande Ospedale Metropolitano, GOM, AO Bianchi Melacrino Morelli, 89124 Reggio Calabria, Italy; massimo.rao@libero.it; 2 Cardiology Unit, ASST Grande Ospedale Metropolitano Niguarda, 20162 Milano, Italy; fabri.oliva@gmail.com; 3 Cardiology Department, Cannizzaro Hospital, 95126 Catania, Italy; simogiub@hotmail.com; 4 Operative Unit of Cardiology, P. Borsellino Hospital, Marsala, ASP Trapani, 91016 Erice, Italy; maur.abri60@gmail.com; 5 Arrhytmia Unit, Division of Cardiology, Ospedale San Paolo, Azienda Sanitaria Locale 2, 17100 Savona, Italy; 6 Sant’Andrea Hospital, ASL 5 Regione Liguria, 19124 La Spezia, Italy; 7 Cardiology Unit, Giovanni Paolo II Hospital, 97100 Lamezia, Italy; doctstefania@hotmail.com (S.A.D.F.); furio.colivicchi@gmail.com (F.C.); 8 Clinical and Rehabilitation Cardiology Department, San Filippo Neri Hospital, ASL Roma 1, 00135 Roma, Italy; roberto_ceravolo@yahoo.it; 9 Cardiology Department, Mauriziano Hospital, 10128 Torino, Italy; irisparrini@libero.it; 10 Cardiology Unit, Città di Castello Hospital, 06012 Città di Castello, Italy; adriano.murrone@gmail.com; 11 Cardiology Department, Sant’Antonio Abate Hospital, ASP Trapani, 91100 Erice, Italy; giovannageraci@hotmail.com; 12 Cardiovascular Department, Sant’Anna e San Sebastiano Hospital, 95122 Caserta, Italy; carminericcio8@gmail.com; 13 Cardiovascular Research Institute, Maastricht University, 6211 LK Maastricht, The Netherlands; sandro.gelsomino@gmail.com; 14 Cardiology Department, F. Miulli Hospital, Acquaviva delle Fonti, 70021 Bari, Italy; m.grimaldi@miulli.it; 15 Cardiology Department, Garibaldi Nesima Hospital, 95122 Catania, Italy; michele.gulizia60@gmail.com

**Keywords:** direct oral anticoagulants (DOACs), perioperative management, reversal agents

## Abstract

A notable increase in direct oral anticoagulant (DOAC) use has been observed in the last decade. This trend has surpassed the prescription of vitamin K antagonists (VKAs) due to the absence of the need for regular laboratory monitoring and the more favorable characteristics in terms of efficacy and safety. However, it is very common that patients on DOACs need an interventional or surgical procedure, requiring a careful evaluation and a challenging approach. Therefore, perioperative anticoagulation management of patients on DOACs represents a growing concern for clinicians. Indeed, while several surgical interventions require temporary discontinuation of DOACs, other procedures that involve a lower risk of bleeding can be conducted, maintaining a minimal or uninterrupted DOAC strategy. Therefore, a comprehensive evaluation of patient characteristics, including age, susceptibility to stroke, previous bleeding complications, concurrent medications, renal and hepatic function, and other factors, in addition to surgical considerations, is mandatory to establish the optimal discontinuation and resumption timing of DOACs. A multidisciplinary approach is required for managing perioperative anticoagulation in order to establish how to face these circumstances. This narrative review aims to provide physicians with a practical guide for DOAC perioperative management, addressing the most controversial issues.

## 1. Introduction

In recent years, the use of direct oral anticoagulants (DOACs) has increasingly risen in venous thromboembolism (VTE) prophylaxis and treatment and stroke prevention in patients with atrial fibrillation (AF), exceeding vitamin K antagonist (VKA) prescription because they do not require routine laboratory monitoring and have a better efficacy–safety profile [1].

Several studies have shown that approximately a quarter of patients on DOACs will require surgery or invasive procedures over two years [2].

Although several guidelines and recommendations on DOAC interruption timing for patients undergoing surgery or interventional procedures have been provided by scientific societies, managing these patients remains debated, and how to obtain a safe and effective approach remains challenging, requiring a personalized evaluation. Even if their short half-life and predictable pharmacokinetics should simplify their periprocedural use, patients receiving DOACs require an appropriate evaluation based on individual patient thrombotic and bleeding risk, procedure-specific bleeding risk, urgency of intervention, and specific DOAC type.

Considering all these factors, it will be necessary to evaluate the case singularly with a multidisciplinary approach on whether or not to withdraw anticoagulation. If OAC needs to be temporarily stopped, when to do it and for how long, whether to perform bridge, whether to carry out specific laboratory tests, and whether and how to use reversal agents are important elements to consider [3,4].

This study aims to present a methodical approach for managing patients receiving DOACs during the perioperative phase.

## 2. Patient-Relevant Parameters

Roughly 25% of patients undergoing oral anticoagulant (OAC) therapy are expected to undergo a surgical or invasive procedure within two years [5,6]. According to the latest guidelines [5,7], when surgery is deemed necessary for patients undergoing OACs, various factors must be assessed, including the nature of the procedure, patient features, and the specific drug(s) used. It is crucial to meticulously evaluate and balance how to deal with these patients in the perioperative period, particularly focusing on the simultaneous ischemic and bleeding risks. In this sense, patient-specific factors encompassing age, gender, individual thrombotic risk, history of bleeding incidents, individual hemorrhagic risk, renal function, comorbidities, and concurrent therapies play an important role [8].

## 3. Age and Gender

Age significantly affects thrombotic risk, serving as a potent driver. This age-related risk increases in individuals aged 65 years and beyond [9]. Moreover, advanced age contributes to the accumulation of DOACs [5].

Gender is an age-dependent modifier of thrombotic risk. Notably, female sex increases the risk of stroke associated with AF [1,10,11]. In the PAUSE trial, female sex has been associated with higher DOAC levels in patients who underwent high-risk surgical procedures [12].

## 4. Individual Thrombotic Risk

AF has been shown to raise the risk of stroke by five times [13,14].

To assess and quantify thrombotic risk in patients with AF, using CHA2DS2-VASc clinical score has been recommended (class I, level A) [7,14]. Assessing parameters such as troponin, natriuretic peptides, von Willebrand factor, C-reactive protein, proteinuria, and D-Dimer could be helpful in evaluating stroke risk in subjects categorized as at low-risk, including those with a single non-sex CHA2DS2-VASc risk factor [15,16]. Obesity [17], hyperlipidemia [18], metabolic syndrome [19], smoking, sleep apnea [20], hypertrophic cardiomyopathy (HCM) [21], amyloidosis (CA) [22] and Anderson–Fabry disease (AFD) [23] have been shown to be associated with a greater thrombotic risk. Moreover, echocardiographic parameters, including left atrium (LA) dilatation, spontaneous echo-contrast or thrombus in LA, low LA auricle velocities, and complex aortic plaque, have been recognized as thrombotic risk factors [24,25]. However, it is important to note that incorporating these factors does not enhance the predictive value of CHA2DS2-VASc [7,26].

**Figure 1:**
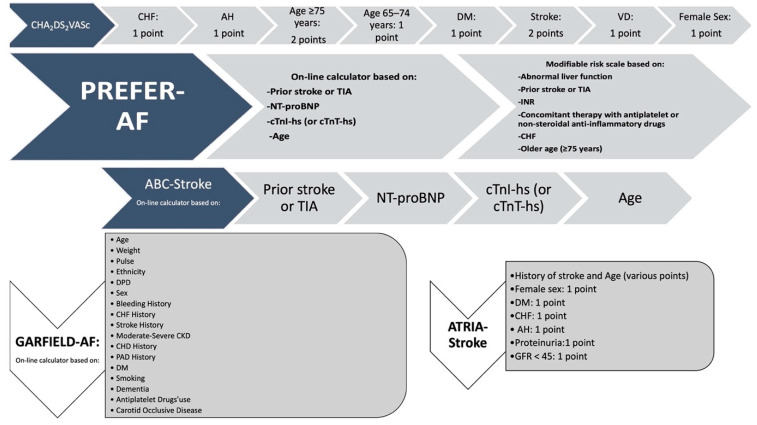
Main clinical scores for arterial thromboembolic risk. CHF: congestive heart failure; AH: arterial hypertension; DM: diabetes mellitus; VD: vascular disease; TIA: transient ischemic attack; NT-proBNP: N-terminal pro–B-type natriuretic peptide; cTnI-hs: high-sensitivity cardiac troponin; INR: labile international normalized ratio; DBP: diastolic blood pressure; CKD: chronic kidney disease; CHD: coronary heart disease; PAD: peripheral artery disease; GFR: glomerular filtration rate.

Additional clinical scores, such as GARFIELD-AF and PREFER-AF, have been proposed to assess thrombotic risk [27,28]. Specifically, those that include biomarkers such as ATRIA [29] and ABC-stroke scores (Figure 1) [30] significantly improve stroke risk prediction [7].

**Figure 2:**
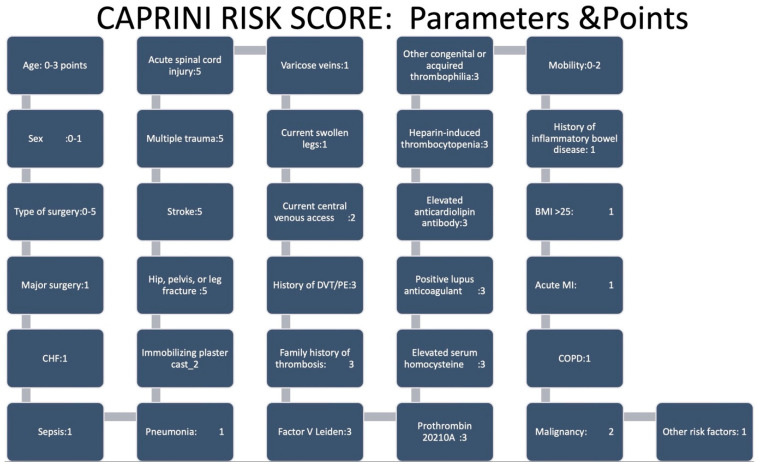
Caprini risk score for venous thromboembolic risk in surgical settings. CHF: congestive heart failure; DVT/PE: deep vein thrombosis/pulmonary embolism; BMI: body mass index; MI: myocardial infarction; COPD: chronic obstructive pulmonary disease.

Regarding perioperative VTE, it is linked to a significant mortality rate, approximately around 17%. Conditions such as recent acute myocardial infarction (AMI), heart failure (HF), acute renal insufficiency, and postoperative infection have been reported to be risk factors for postoperative VTE [31]. However, its incidence is unclear, although it is likely to be underestimated because the reliability of screening and diagnostic tools (such as D-dimer and symptoms) is poor in this context [31]. With this aim, the Caprini score (Figure 2) has been introduced to assess the risk of VTE in various surgical contexts. According to this score, performing a thromboprophylaxis strategy should be considered in the presence of moderate and high values, >5–8 points and ≥9 points, respectively [32]. Patients who have experienced a thromboembolic accident during a previous interrupted DOAC strategy should be regarded as being at a high thrombotic risk [5]. Moreover, the occurrence of a recent stroke (within three months), AF with a CHA2DS2-VASc score > 6, the presence of LV apical thrombus, deficiency of antithrombin III or protein C and/or S have been associated with a very high thromboembolic risk [5].

In the PAUSE trial [12], elevated DOAC levels have been associated with a body weight < 70 kg and creatinine clearance (CCl) < 50 mL/min among patients undergoing high-risk surgery.

## 5. History of Bleeding Complications and Individual Hemorrhagic Risk

It is crucial to identify the underlying predisposing pathology for patients who have recently experienced a bleeding complication. In gastrointestinal bleeding, the potential guilty conditions should be accurately investigated [7].

Moreover, other bleeding risk factors, such as an advanced age (>65 years), previous major bleeding (MB) or stroke, chronic kidney disease (CKD) and chronic liver disease (CLD), cancer [33], genetic conditions including CYP 2C9 polymorphisms, diabetes mellitus, (DM), and cognitive impairment, play a substantial role in contributing to bleeding events. Additionally, conditions such as extreme frailty, anemia and thrombocytopenia, arterial hypertension (AH), concomitant drug use, and excessive alcohol intake increase hemorrhagic risk [34].

Furthermore, in patients on OACs with a history of falls, although they need a careful evaluation due to their frailty, this element has not been considered an independent predictor of bleeding [35].

Importantly, it is essential to consider the role of the overall assessment of bleeding risk [7].

**Figure 3:**
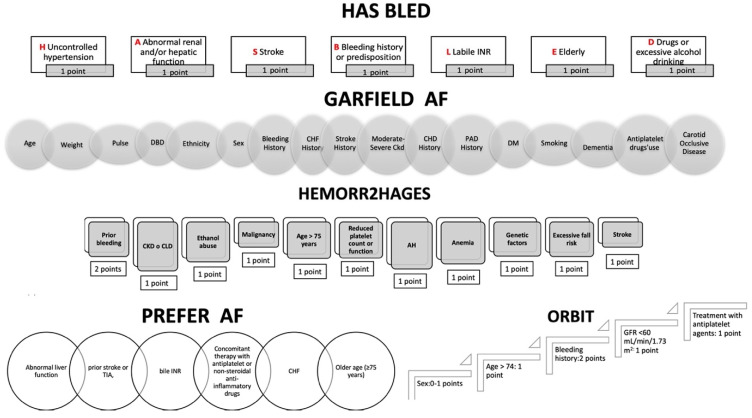
Main clinical scores for bleeding risk. INR: labile international normalized ratio; DBP: diastolic blood pressure; CHF: congestive heart failure; CHD: coronary heart disease; PAD: peripheral artery disease; DM: diabetes mellitus; CKD: chronic kidney disease; CLD: chronic liver disease; AH: arterial hypertension; TIA: transient ischemic attack; GFR: glomerular filtration rate.

Indeed, taking into account exclusively modifiable bleeding risk factors was an ineffective approach [36,37,38]. On the contrary, a more complete evaluation based on a standardized score system, including modifiable and non-modifiable factors, has been recommended (Class I level B) [7,13,29,39,40,41]. Several bleeding risk scores have been proposed [13,27,28,42,43,44] (Figure 3). However, refs. [13,27,28,42,43,44] data are conflicting [28,44,45] and, by and large, they have been considered poorly predictive for hemorrhagic events [46]. However, according to data from 38 studies [39], the HAS-BLED score was confirmed to be the most reliable for predicting bleeding risk, comparing bleeding risk prediction approaches [47].

Nonetheless, according to other data, its accuracy seems to be scarce. Moreover, it has been argued that it has been performed in cohorts of patients with low hemorrhagic complications [13,48,49], and what is more, HAS-BLED has been validated in cohorts of patients on VKAs. In contrast, most patients assume DOACs [50]. Indeed, utilizing a risk score that assesses the quality of warfarin anticoagulation, considering the labile international normalized ratio (INR) as a parameter, may be less reliable for patients who are taking DOACs.

Conversely, the DOAC Score has been developed to categorize the hemorrhagic risk in individuals on DOACs [51].

This tool stands out for its ability to differentiate bleeding risk across a broader range of age groups and different classes of renal function [52,53], resulting in more accuracy compared to previous scoring systems. Notably, unlike other scores, the DOAC risk score has been specifically developed for patients assuming DOACs [51].

Finally, this score also takes into account the cumulative risk of polytherapy.

It is noteworthy that numerous biomarkers, along with certain clinical risk factors, have predictive value for both stroke and bleeding [7,30]. Accordingly, studies such as GARFIELD and PREFER-AF predict both thrombotic and bleeding events.

Moreover, the GARFIELD-AF bleeding score has been introduced as a predictor of major bleeding in AF patients [27]. Importantly, it has a poor predictive capability for bleeding outcomes and none in predicting bleeding outcomes. On the contrary, if the HAS-BLED score also has a modest capability of predicting bleeding, it strongly correlates with bleeding outcomes [54].

The ABC-bleeding risk score, which takes into account age, biomarkers, and clinical history data [55], seemed very reliable. It is important to highlight that this score may support decisions regarding the appropriate combination and duration of dual antithrombotic therapy (DAT) in patients with AF and coronary artery disease (CAD) necessitating both OAC and antiplatelet drugs based on the bleeding risk [51,56].

The ORBIT score, developed from data from the ORBIT-AF registry community-based population based on only five bleeding factors, kept up with HAS-BLED and ATRIA scores, and it seems to have a broader applicability than existing bleeding scores [44].

HAS-BLED has been demonstrated to perform better than other scores such as HEMORR2HAGES [42] and ATRIA [43], especially in terms of intracranial hemorrhage prediction [48]. However, other studies did not confirm a long-term advantage over the HAS-BLED score [13]. Thus, according to ESC guidelines (class IIa, level B) [7], the HAS-BLED score is the recommended score for assessing hemorrhagic risk [13,19,28,30].

## 6. Renal Function

Irrespective of AF, CKD is a medical condition that can contribute to both a prothrombotic and hemorrhagic state, favoring a hypercoagulability or hemorrhagic condition, respectively [57].

CKD is known to favor DOAC accumulation. In the PAUSE trial [12], subjects with a CCl value inferior to 50 mL/min exhibited elevated levels of DOACs during high-risk surgery [5,7].

## 7. DOAC in Chronic Advanced Liver Disease

Advanced CLD may lead to alterations in the balance of pro- and anti-thrombotic factors, with a consequent increased risk of thrombosis or bleeding [58]. Furthermore, patients with CLD may need anticoagulants to treat or prevent venous thromboembolic events or prevent stroke and systemic thromboembolism in the case of AF. In clinical practice, using anticoagulant agents in these patients is a challenge. As regards DOACs, patients with significant CLD and upper limit normal values (ULN) of hepatic enzymes (HE) (specifically HE> >2 × ULN for apixaban, dabigatran, and edoxaban, >3 ULN for rivaroxaban) were excluded from a phase three RCT that tested these agents both in AF and in venous thromboembolism (VTE). As a consequence, DOACs are currently contraindicated in this specific setting. In more detail, apixaban, dabigatran, and edoxaban must be used with caution in patients with Child–Pugh B liver disease and avoided in Child–Pugh C. In contrast, rivaroxaban is contraindicated in patients with Child–Pugh B and C. Observational studies provide some evidence of DOAC use in advanced liver disease. The findings from these investigations indicate that the risk of bleeding associated with DOACs is comparatively lower when juxtaposed with that of warfarin or LMWH [58]. In patients who need invasive procedures, withdrawing DOAC based on standard recommendations [2] allows them to avoid exposure to an increased procedure-related bleeding risk [59]. In light of the elevated susceptibility to spontaneous bleeding, the administration of anticoagulant therapy necessitates careful consideration, with due regard to the individual’s bleeding–thrombosis risk ratio. Although common laboratory coagulation tests do not allow an accurate estimation of the hemorrhagic risk associated with LCD and DOAC treatment, if invasive procedures are planned or in case of active bleeding, these tests may provide information on the patient’s hemostatic status. They can help choose therapeutic measures to implement. A multidisciplinary team should establish this choice [60].

## 8. Cardiovascular Comorbidities

Peripheral arterial disease (PAD) and AMI have been correlated with a heightened thrombotic risk ranging from 17% to 22%, particularly notable among individuals of Asian ethnicity [36]. The evidence of significant CAD and complex aortic plaque have been recognized as independent risk factors for ischemic stroke in AF subjects [40,61]. Moreover, those who underwent a mechanical aortic valve replacement (AVR) run a consistent thrombotic risk, particularly in the case of an older-generation mechanical aortic prosthesis or a mechanical mitral or tricuspid valve replacement [5,62,63].

Furthermore, lifelong OAC therapy may be required in patients with congenital heart disease (CHD), primarily due to arrhythmias. It is important to note that the CHA2DS2-VASc score, commonly used for risk assessment in AF, has not been validated for these patients [5].

## 9. Non-Cardiovascular Comorbidities

Cancer has been shown to be a condition that increases both thrombotic and hemorrhagic risk [5,64,65], so that cancer patients typically require a multidisciplinary team approach involving careful decision making [66]. Various factors should be considered in this sense, including the type of cancer, its site(s), staging, and specific cancer therapies [5,7,67]. Moreover, it should be noted that the scores commonly used, such as CHA2DS2-VASc and HAS-BLED, are not specific for cancer patients [7]. Moreover, it is worth noting that certain gastrointestinal conditions, such as active inflammatory bowel disease, can raise the risk of stroke [68].

CLD occurrence poses significant challenges, as it is associated with both a hemorrhagic and thrombotic risk, requiring specific consideration and a multidisciplinary approach [69].

Anemia is an independent predictor of MB events and must be carefully investigated in anticoagulated patients [30,44].

## 10. Concomitant Therapies

Dual antithrombotic therapy (DAT), consisting of the combined use of OAC and anti-platelet drugs, is known to increase bleeding risk [44]. This approach is required when percutaneous coronary intervention (PCI) is performed in AF patients [7]. Furthermore, patients who have undergone a recent transcatheter aortic valve implantation (TAVI) may also be candidates for DAT. In these cases, the choice and duration should be individualized [56,70].

Importantly, certain treatments may interact with DOACs [5]. Indeed, the administration of amiodarone, verapamil, or diltiazem was linked to increased pre-operative levels of DOACs, as observed in the CORIDA trial [71].

## 11. Urgent Unplanned Procedures in Patients on DOACs

Urgent procedures require immediate action, usually within hours. Surgery or invasive procedures should be postponed whenever feasible until at least 12 h, preferably 24 h, after the last DOAC assumption.

In particular, when postponing the procedure could result in more unfavorable outcomes than an immediate intervention, or when the last DOAC dose is unknown, it may be prudent to await coagulation test results. This would facilitate an assessment of the necessity for specific reversal agents or the utilization of prothrombin complex concentrates (PCCs).

Acute emergency procedures need to operate within minutes and cannot be delayed. In patients requiring emergency surgery, reversal with idarucizumab for dabigatran and andexanet alfa (“off-label”) for FXa inhibitors is advisable, especially in moderate-to-high hemorrhagic risk procedures or when surgery is required for ongoing bleeding (e.g., spontaneous bleeding or major trauma) [72]. Idarucizumab 5 g divided into two 2.5 g intravenous doses, given up to 15 min apart, has been shown to achieve normal hemostasis within minutes among patients on dabigatran undergoing urgent procedures [50].

Andexanet alfa neutralizes the anti-Xa DOACs, and thus should swiftly reduce the anti-factor Xa activity [51]. The onset of efficacy is observed within two minutes after administering the IV bolus dose. The attenuated anti-factor Xa activity persists for up to two hours after the cessation of the infusion. Furthermore, it is noteworthy that andexanet alfa can inhibit tissue factor pathway inhibitor, with this inhibition sustained for a minimum of 22 h post-administration of andexanet alfa [72].

Under circumstances where dedicated reversal agents are unavailable, activated prothrombin complex concentrate (aPCC) and prothrombin complex concentrate (PCC) may be considered alternatives, notwithstanding the limited evidence regarding their efficacy and safety [72]. The supportive data for the utilization of PCCs in patients undergoing DOACs with major bleeding episodes or necessitating urgent surgical interventions are scant and predominantly drawn from retrospective analyses. For patients on DOACs with anticipated or confirmed clinically significant anticoagulant levels, an initial intravenous dose of 50 U/kg is recommended. In instances where reversal agents are unavailable or not utilized, it is advisable to opt for immediate and urgent procedures conducted under general anesthesia as opposed to spinal anesthesia. This precautionary measure aims to mitigate the potential risk of spinal hematoma.

## 12. Reversal Agents

Idarucizumab is a humanized monoclonal antibody fragment that binds to dabigatran with an affinity 350 times greater than thrombin. In the REVERSE AD study [73], idarucizumab was effectively employed in dabigatran-treated patients with severe bleeding or requiring urgent surgery within 8 h, showing a safe and effective profile.

Andexanet alfa is an antidote for FXa inhibitors. It is a modified recombinant FXa, enzymatically rendered inactive through a mutation in its catalytic site, which, however, has retained the ability to bind direct and indirect FXa inhibitors [72]. This allows it to sequester anticoagulants within the vascular space and neutralize their effect [72].

In the ANNEXA-4 study [74], this agent reduced anti-FXa activity by 92%, 92%, and 75% for apixaban, rivaroxaban, and enoxaparin, respectively, allowing an excellent or good hemostasis in 82% of the treated population.

Unlike idarucizumab, andexanet alfa has not been evaluated for reversing anti-FXa activity before urgent surgery. Moreover, specific studies do not exist for PCC/aPCC in this context [72].

Hence, in situations where the patient’s life or the procedure is at risk, considering andexanet alfa or PCC/aPCC becomes crucial. It is important to note that the reversal agents may induce a prothrombotic rebound, necessitating multidisciplinary management to determine the optimal timing for resuming OAC [72].

In both the RE-VERSE AD [73] and ANNEXA-4 [74] studies, incidences of thrombotic events manifested in 4.8% and 10% of patients, respectively, within one month.

Establishing whether thrombotic events result from the intrinsic procoagulant effect of reversal agents or the prothrombotic profile associated with patients’ comorbidities, heightened by conditions including inflammation, immobility, or blood transfusions, is a considerable challenge. No prothrombotic effects have been associated with idarucizumab use; however, in patients treated with andexanet alfa, a temporary increase in D-dimer levels has been reported [72].

## 13. Planned Interventions in Patients on DOACs

**Figure 4:**
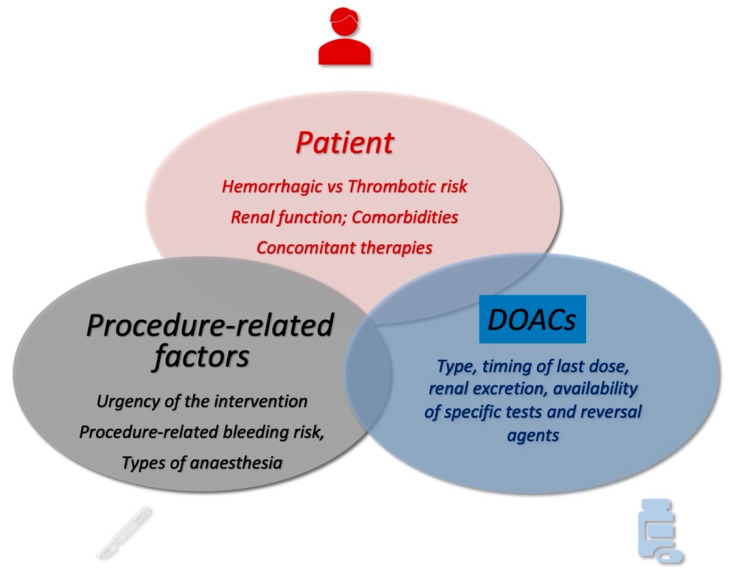
Key factors to take into consideration in the perioperative management of DOACs.

If the procedure can be planned, the timing of the last dose of DOAC before starting an elective intervention should be determined according to DOAC type (dabigatran vs. FXa inhibitors), procedural bleeding risk (low vs. high bleeding risk), and the patient’s renal function based on creatinine clearance (CrCl) ranges of ≥80, 50 to 79, 30 to 49, and 15 to 29 mL/min [5] (Table 1, Figure 4). Notably, the bleeding risk needs to be carefully evaluated [75,76,77,78].

A temporary DOAC interruption or no interruption may be adopted for many minimally invasive procedures with a low bleeding risk.

Preceding surgical intervention, particularly in cases posing a minimal risk of bleeding, the administration of DOAC therapy should be halted for a duration of one day before the procedure, accounting for an interval ranging from 36 to 42 h, which approximately aligns with three half-lives of DOACs. Conversely, in scenarios involving a heightened risk of bleeding, cessation of DOAC therapy should occur two days before the scheduled procedure, spanning an interval of 60 to 68 h, which corresponds to approximately five half-lives of DOACs. Notably, individuals undergoing dabigatran treatment with CrCl levels below 50 mL/min necessitate extended discontinuation intervals to accommodate the renal-dependent clearance of dabigatran.

Table 2 summarizes the recommended timing for the last intake of DOACs before planned procedures.

It is important to note that the existing evidence on perioperative DOAC management is focused on patients with AF [79,80,81,82,83,84,85,86].

Only a few studies, primarily retrospective case series, have investigated perioperative DOAC management in patients with VTE [87,88].

The main studies investigating perioperative DOAC interruption for elective procedures in patients with AF are summarized in Table 3.

When evaluating studies that have examined the discontinuation of DOACs in patients with AF for elective procedures, it is crucial to consider the limitations of these studies and potential biases. None of the RCTs had pre-defined perioperative analyses or postoperative outcome definitions before conducting such assessments. As a result, the reported pooled estimates may be biased and underestimate the true rate of complications associated with DOAC discontinuation within 30 days. However, these post hoc analyses incorporate widely accepted outcome definitions based on standard 30-day complication rates, which helps mitigate bias. Furthermore, there was significant variability in perioperative anticoagulation practices among different studies. Only a few studies had well-defined protocols for perioperative anticoagulation that provided clear instructions on when to stop and restart anticoagulation in the context of an invasive procedure. The rates of bridging anticoagulation with alternative parenteral anticoagulation also varied significantly across studies. Overall, the bridging rates were low but ranged between 4.5% and 17.0% for patients on DOACs. The timing and dosage of bridging anticoagulation (i.e., preoperative, perioperative, or postoperative only) are also unknown.

Finally, most studies do not provide results based on the specific type of procedure. The risk of these clinical outcomes is likely to differ by procedure, as the risks of bleeding and thromboembolism vary considerably across different procedures.

Dental surgery is generally considered a low-bleeding-risk procedure with the potential for effective local hemostasis. While most guidelines for dental surgery recommend continuing the use of DOACs, these recommendations often rely on limited evidence. Dental extraction can typically be safely performed in an outpatient setting, utilizing local hemostatic measures without interrupting anticoagulation. This can be achieved by conducting the procedure at the trough level or by skipping the morning dose of the DOAC [89,90,91].

In patients with CCS (chronic coronary syndrome) undergoing elective PCI and receiving DOAC treatment, DOAC should be discontinued at least 24 h before the last intake, before patients are taken to the cardiac catheterization lab. Periprocedural anticoagulation should be used per local practice. Unfractionated heparin (70 IU/kg) rather than LMWH is preferred. Unfractionated heparin should be administered to target ACT or aPTT levels per standard clinical practice. After discontinuation of parenteral anticoagulation, DOAC should be restarted in combination with antiplatelet therapy [2].

Epidural anesthesia, spinal anesthesia, and lumbar puncture are procedures that necessitate normal blood clotting function and are categorized as ‘high risk’. Current guidelines discourage the use of neuraxial anesthesia or deep blocks when patients are on uninterrupted DOACs. Instead, the recommendation is to discontinue DOACs for a specified period, typically up to five half-lives (3 days for factor Xa inhibitors and 4–5 days for dabigatran) [92,93].

After undergoing procedures such as epidural anesthesia, spinal anesthesia, or lumbar puncture, DOACs can typically be resumed 24 h afterward. However, procedures such as peripheral nerve blocks or injections into peripheral joints and musculoskeletal areas may not necessarily require the interruption of DOACs. If interruption is considered necessary for these low-risk procedures, it is typically for a shorter period, such as two half-lives [93].

In conclusion, each medical institution should assemble a collaborative, multidisciplinary team comprising experts in thrombosis and hemostasis. This team should include professionals such as laboratory specialists, anesthesiologists, intensivists, endoscopists, and surgeons representing diverse specialties. The primary objective of such a team would be to delineate and establish the most effective perioperative management protocols tailored specifically for patients undergoing DOAC therapy.

## 14. Interventions with High Bleeding Risk and Increased Thromboembolic Risk: Cardiac Ablation Procedure

Catheter ablation (CA) procedures performed in the left atrium (LA) or left ventriculum (LV) present a rather unique situation, encompassing risks of both significant bleeding and thrombotic events. According to the latest international guidelines, CA of AF should proceed without interruption of DOACs [7,60,94,95], and it is mandatory to utilize unfractionated heparin (UFH) before or immediately after the trans-septal puncture to reach and maintain a target activated clotting time (ACT) of at least ≥300 s [94]. As assessed in several studies [96], in patients with uninterrupted DOAC, often a greater amount of UFH is needed to reach the target of ACT ≥ 300 s, and the administration of a higher amount of heparin was associated with an increased rate of procedural major bleedings.

Various studies support the uninterrupted administration of DOACs during CA of AF [97]. The RE-CIRCUIT study [98] revealed a notably reduced occurrence of major bleeding in patients on uninterrupted dabigatran compared to those on uninterrupted warfarin, with no significant disparities in strokes or other thromboembolic events. Similarly, in the VENTURE-AF trial, individuals undergoing CA for AF on uninterrupted rivaroxaban exhibited a decreased frequency of hemorrhagic and thrombotic events compared to those on VKAs [99].

Moreover, AXAFA-AFNET 5 [100] and ELIMINATE-AF trials [101] have shown that an uninterrupted regimen of apixaban and edoxaban, respectively, is superior in terms of safety and efficacy to uninterrupted VKA therapy.

Nevertheless, this approach increases the risk of bleeding complications, especially vascular ones [102]. Therefore, it is essential to employ vascular ultrasound for femoral vein puncture to reduce vascular complications, as supported by relevant studies [103,104].

## 15. Bridging and DOAC Restarting

The predictable decrease in DOACs’ anticoagulation effect allows for well-timed temporary discontinuation of DOAC treatment before surgery without bridging.

In the PAUSE trial, AF patients who were enrolled and subsequently underwent elective surgery exhibited decreased thrombotic and bleeding events when DOAC discontinuation was implemented without the adjunctive use of heparin bridging [86].

In the Dresden DOAC registry, in patients on DOACs referred for invasive procedures, the rate of MACE was comparable to that of those who underwent bridging strategy, no bridging, or no OAC discontinuation [6]. Although bridging has not been recognized as a bleeding-independent risk factor, this approach has been adopted, especially in the case of major procedures resulting in a great likelihood of major bleeding.

Consequently, pre-operative bridging with low-molecular-weight heparin (LMWH) or UFH is not advised for patients taking DOACs.

Exceptions may arise in very few high-risk scenarios, such as urgent surgeries with a high bleeding risk for patients who have recently experienced a thromboembolic event (<3 months) or those who had an event during a previous interruption of DOAC therapy. Besides timing DOAC interruption, a multidisciplinary team might consider switching to UFH or low-dose dabigatran.

After surgery, DOAC resumption depends on the patient’s hemostasis.

In many cases, in stabilized patients, DOACs can be restarted 6–8 h after the procedure. In high-bleeding-risk procedures or in patients unable to take oral drugs, consideration should be given to the use of LMWH 6–8 h after the procedure, delaying the resumption of DOACs for 48–72 h.

## 16. Laboratory Testing before and after Surgery

DOACs have demonstrated predictable pharmacokinetics and pharmacodynamics, characterized by a wide therapeutic range. As a result, routine laboratory monitoring of their anticoagulant effect is deemed unnecessary. Nevertheless, in scenarios necessitating emergent or urgent procedures where specific antidotes are required, knowing the anticoagulation status of patients taking DOACs may prove beneficial.

In such contexts, basic coagulation tests routinely performed in all laboratories, such as prothrombin time (PT), activated partial thromboplastin time (aPTT), or thrombin time (TT), are scarcely sensitive. For dabigatran, aPTT provides a qualitative assessment of anticoagulant activity (present/absent) but lacks sensitivity in quantitative terms. Dabigatran has less impact on PT. Therefore, measuring this coagulation parameter is not useful. Rivaroxaban, apixaban, and edoxaban affect PT to varying degrees. Due to the variability in PT prolongation and its dependence on liver function, this parameter can only offer a qualitative assessment of anticoagulant activity. Specific quantitative coagulation tests for DOACs, not readily available in all laboratories, include diluted thrombin time (dTT) and ecarin clotting time (ECT) for dabigatran and DOAC-calibrated chromogenic anti-Xa activity assays for FXa inhibitors [105,106].

In clinical practice, coagulation test results must be interpreted cautiously, considering the timing of the last DOAC intake. Moreover, neither the results of coagulation tests nor plasma levels of anticoagulants can serve as definitive criteria for using antidotes or nonspecific reversal agents [3].

## 17. How and When to Restart DOACs after Invasive Procedures or Surgery

In uncomplicated procedures with the achievement of stable and complete hemostasis, OAC with DOAC or LMWH can be resumed 6–8 h after the end of the intervention [107]. However, in certain surgical procedures (e.g., neurosurgery, procedures with challenging hemostasis), restarting full-dose anticoagulation within the first 48–72 h may not have a favorable risk–benefit ratio, as the postoperative bleeding risk could be higher than the thrombotic risk for which anticoagulation would be required. In these cases, a prophylactic dose of LMWH could be considered, and the resumption of OAC could be delayed by 48–72 h. Therefore, assessing each case regarding individual ischemic and hemorrhagic risk is recommended. The timing of resuming OAC therapy in the postoperative period may also be delayed in the presence of clinically significant hemorrhagic complications.

**Figure 5:**
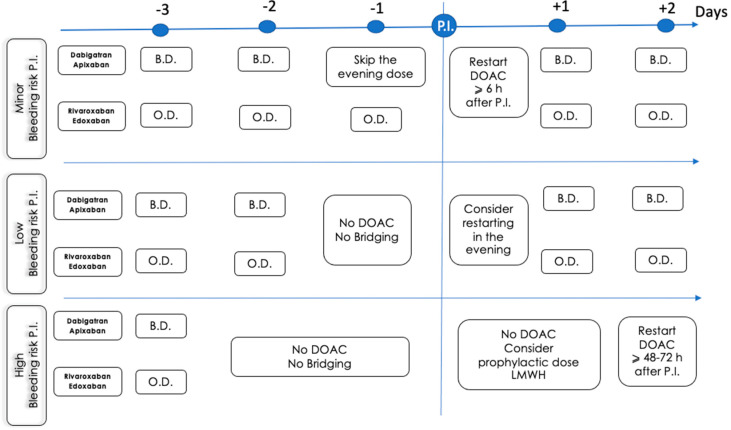
Protocols for stopping and restarting DOAC therapy in planned interventions. Modified from Halvorsen S. et al. ESC Guidelines EHJ 2022 [5]. P.I.: planned intervention, O.D.: once daily; B.D.: twice daily; LMWH: low-molecular-weight heparin, DOAC: direct oral anticoagulant.

Figure 5 illustrates the protocols for stopping and restarting DOAC therapy according to the bleeding procedural risk, the specific type of DOAC, and its method of administration in planned interventions.

## 18. Combination Therapy (Antiplatelets and Anticoagulants)

Patients taking OAC may also require antiplatelet therapy. Indeed, concomitant conditions, such as AF, VTE, and the presence of mechanical valves, occur in about 5–10% and 6–8% of patients with CAD and acute coronary syndrome (ACS), respectively [108]. Moreover, those who underwent PCI may require triple antithrombotic therapy (TAT) for a short period of time (usually seven days) based on their ischemic and bleeding risk. After discontinuing TAT, these patients have to continue double antithrombotic therapy (DAT) consisting of OAC combined with a single antiplatelet drug for up to one year. In case of very high ischemic risk (e.g., prior stent thrombosis while in OAC, PCI of bifurcation lesions with two stents implanted, treatment of chronic total occlusion, total stent length > 60 mm), DAT may be considered for more than one year. For patients on OAC requiring PCI, it remains uncertain whether bridging VKA or DOACs with parenteral anticoagulants or maintaining OAC alone is safer. Among patients requiring urgent PCI, recent guidelines suggest performing PCI without interruption of VKAs or NOACs to reduce the risk of bleeding [109]. In VKA-treated patients, additional parenteral anticoagulation is unnecessary when the INR exceeds 2.5. In patients on DOACs, regardless of the timing of the last administration of DOACs, add low-dose parenteral anticoagulation (e.g., enoxaparin 0.5 mg/kg i.v. or unfractioned heparin 60 IU/kg).

Pivotal randomized clinical trials evaluating antithrombotic strategies among patients with AF undergoing PCI are summarized in Table 4. Overall, in patients with AF without mechanical prosthetic valves or moderate to severe mitral stenosis, the evidence supports the use of DOACs over VKAs, as they reduce bleeding risk. DAT with a DOAC at the recommended dose for stroke prevention and single antiplatelet agent (SAPT) (preferably clopidogrel, which was used in >90% of patients in the major RCTs) is recommended as the default strategy for up to 12 months after up to 1 week of TAT (with DOAC and DAPT consisting of aspirin and clopidogrel). The suggested one-week duration of TAT is based on the median treatment duration in the investigational arm of the AUGUSTUS trial. While prasugrel or ticagrelor have been evaluated in a minority of ACS patients, their use within TAT is not recommended due to the lack of robust safety and efficacy data, and clopidogrel should be the P2Y12-i of choice. Although none of the available RCTs were designed to detect differences in ischemic events, the numerically higher risk of stent thrombosis and MI is offset by the lower risk of bleeding, with a resultant neutral effect on total mortality.

In case of unplanned surgery that cannot be deferred, the interruption of antithrombotic therapy should be evaluated case by case, taking into account the bleeding and the ischemic risk. A multidisciplinary approach involving cardiologists, surgeons, and anesthesiologists is warranted. Procedural and individual factors determine the bleeding risk. Previous consensus documents endorsed by scientific societies may help evaluate this risk by classifying surgical procedures into three categories: low, intermediate, and high bleeding risk [110]. The ischemic risk is derived by patients’ characteristics (e.g., age, diabetes mellitus, acute kidney injury (AKI), atherosclerotic burden), features of the previous PCI (e.g., bifurcation lesions, number of stents implanted, total stent length), and time from the last PCI or ACS. Patients with recent ACS or PCI are at higher risk of ischemic events, especially after the first weeks. Therefore, postponing surgery at least one month after ACS or PCI is recommended if possible. Within 30 days after performing surgery, a multidisciplinary team should carefully evaluate the benefits of ACS or PCI.

It is not recommended to perform surgery on TAT despite these patients having high thrombotic risk. Although data are lacking in this particular scenario, it is likely that the theoretical benefit of TAT in reducing ischemic events will not outweigh the expected increase in bleeding events. OAC can be stopped, as discussed above, depending on the procedure and patients’ characteristics. When a low bleeding risk has been estimated in subjects with ACS or PCI within 30 days, dual antiplatelet therapy (DAPT) should be continued. Conversely, when a moderate/high bleeding risk has been assessed, therapy with a P2Y12 inhibitor should be stopped five days before (7 days for prasugrel), and acetylsalicylic acid (ASA) should continue. In selected patients with particularly high ischemic risk or who need to stop both ASA and P2Y12 inhibitors, bridging therapy with tirofiban or cangrelor could be considered [107,111]. In these cases, OAC should be stopped before starting intravenous antiplatelet.

**Table 4:** Clinical trials evaluating antithrombotic strategies among patients with AF undergoing PCI.

Trial	Study Design	Intervention	Key Findings	Number of Patients Enrolled
PIONEER AF [112]	Randomized, Open-label	Rivaroxaban + DAPT vs. VKA + DAPT vs. Triple Therapy	Reduced bleeding risk with rivaroxaban-based regimen (15 mg or 2.5 mg BID) plus P2Y12 inhibitor compared to triple therapy at 12 months. Similar rates of ischemic events.	2124
RE-DUAL PCI [113]	Randomized, Open-label	Dabigatran + P2Y12 Inhibitor vs. VKA + DAPT	Lower rates of bleeding with dabigatran 110 mg and similar rates with dabigatran 150 mg compared to VKA plus P2Y12 inhibitor and ASA. Comparable rates of ischemic events.	2725
AUGUSTUS [114]	Randomized, Double-blind	Apixaban vs. VKA + P2Y12 Inhibitor + ASA vs. Apixaban + P2Y12 Inhibitor vs. VKA + P2Y12 Inhibitor	Reduced bleeding with apixaban without a significant increase in ischemic events when compared to VKA plus P2Y12 inhibitor and ASA. Lower bleeding with apixaban + P2Y12 inhibitor vs. VKA + P2Y12 inhibitor + ASA.	4614
ENTRUST-PCI [115]	Randomized, Double-blind	Edoxaban + P2Y12 Inhibitor vs. VKA + P2Y12 Inhibitor	Similar rates of clinically relevant bleeding with edoxaban vs. VKA, and non-inferiority of edoxaban regarding composite efficacy endpoint (major adverse cardiac events, thromboembolic events, or all-cause death).	1506

DAPT: dual antiplatelet therapy: VKA: vitamin K antagonist.

## 19. Conclusions

A deep knowledge of the pharmacokinetic and pharmacodynamic mechanisms of DOACs, the appropriateness of their use, and the specific surgical contexts need to be known by physicians in order to detect and face issues promptly.

In elective procedures, DOAC interruption must be performed, balancing DOACs’ half-life, the specific procedure’s bleeding risk, and the thrombotic patient profile. With regards to laboratory tests, considering the great inter-individual variability of plasmatic DOAC values, they may be performed in selected clinical scenarios.

Emergency procedures represent a challenging issue due to bleeding complications. In these cases, the use of antidotes needs to be evaluated. Performing multidisciplinary evaluation in these patients remains the most advisable approach.

## Figures and Tables

**Table 1:** Procedure bleeding risk classification.

Minor Risk	Low Risk	High Risk
Dental extractions, paradontal surgery, edodontic procedure	Complex dental procedures (e.g., multiple tooth extractions)	Neurosurgery
Subgingival scaling or other cleaning	Endoscopy with simple biopsy	Any surgery with neuraxial anesthesia
Cataract surgery	Small orthopedic surgery	Cardiac surgery
Endoscopy without biopsy or resection		Thoracic surgery
Dermatologic procedures		Urological surgery/biopsy
Intramuscular injection		Major orthopedic surgery
Gastroscopy or colonscopy without biopsies		Major intra-abdominal surgery
Coronary angiography		Complex endoscopy
Pacemaker or internal defibrillator placement		Complex cardiological interventions, e.g., lead extraction, epicardial VT ablation, complex PCI

VT: ventricular tachycardia; PCI percutaneous coronary intervention.

**Table 2:** Timing of last DOAC intake before planned interventions.

Renal Function CrCl (mL/min)	Dabigatran		FXa Inhibitors	
	Low Risk	High Risk	Low Risk	High Risk
	No bridging with LMWH/UFH			
≥80	≥24 h	≥48 h	≥24 h	≥48 h
50–79	≥36 h	≥72 h		
30–49	≥48 h	≥96 h		
15–29	Not indicated	Not indicated	≥36 h	
<15	No official indication for use			

Modified from Halvorsen S. et al. [5] ESC Guidelines EHJ 2022. Table 2 legend: CrCl: creatinine clearance, LMWH: low-molecular-weight heparin; UFH: unfractionated heparin.

**Table 3:** Perioperative DOAC interruption for elective procedures.

Study	Study Design	DOAC	Outcome	Intervention and Comparison	RR (95% CI)
RELY TRIAL [79]	RCT	Dabigatran 150	MB	Stopping dabigatran 24 h before low-bleeding-risk procedure and 2–5 days before high-bleeding-risk procedure according to renal function. Resuming dabigatran once adequate hemostasis had been achieved	3.8 (n.a.)
		Dabigatran 110			2.8 (n.a.)
Nakamura R, et al. [81]. Journal of cardiology. 2019	Observational	Dabigatran	MB	Continuing vs. stopping	1.5 (0.55–3.92)
			Thromboembolism		2.2 (1.32–3.8)
Garcia D, et al. [82]	Observational	Apixaban	MB	Continuing vs. stopping	1.8 (0.5–6.7)
			Thromboembolism		0.9 (0.06–15.3)
AEIOU trial [80]	RCT		Stroke		0.9 (0.02–48.4)
ENGAGE AF-TIMI 48 [84]	RCT	Edoxaban	MB	Continuing vs. stopping	2.2 (1.4–3.54)
			Thromboembolism		1.2 (0.6–2.26)
BRUISE CONTROL-2 [84]	RCT	All DOACs	Stroke	Continuing vs. stopping DOACs 1–4 days before surgery/procedure	1.02 (0.06–16.2)
			PE		1.02 (0.02–51.2)
			MI		1.02 (0.02–51.2)
ROCKET AF [85]	RCT	Rivaroxaban	MB	LMWH bridging vs. no bridging during DOAC interruption	1.1 (0.43–2.62)
			Thromboembolism		0.5 (0.06–3.45)
			MI		2.3 (0.82–6.7)
BRUISE CONTROL-2 [84] European heart journal. 2018	RCT	All DOACs	Major bleed	Resuming DOACs ≤ 24 h vs. >24 h after elective procedures	1.02 (0.36–2.9)
			Stroke		1.02 (0.06–16.2)
			MI		1.02 (0.02–51.2)
PAUSE Study [86] JAMA Intern Med. 2019	Prospective cohort design	Dabigatran	MB	Stopping DOACs 1 day before low-bleeding-risk procedure and 2 days before high-bleeding-risk procedure. Resuming DOACs 1 day after a low-bleeding-risk procedure and 2 to 3 days after high-bleeding-risk procedure	0.9 (0–1.73)
			Thromboembolism		0.6 (0–1.33)
		Apixaban	MB		1.35 (0–2.0)
			ATE		0.16 (0–0.48)
		Rivaroxaban	MB		1.85 (0–2.65)
			ATE		0.37 (0–0.82)

MB: major bleeding; PE: pulmonary embolism; MI: myocardial infarction; ATE: arterial thromboembolism; DOAC: direct oral anticoagulant; LMWH: low-molecular-weight heparin.
